# Women’s experiences of decision-making and informed choice about pregnancy and birth care: a systematic review and meta-synthesis of qualitative research

**DOI:** 10.1186/s12884-020-03023-6

**Published:** 2020-06-10

**Authors:** Cassandra Yuill, Christine McCourt, Helen Cheyne, Nathalie Leister

**Affiliations:** 1 Centre for Maternal and Child Health Research, School of Health Sciences, City, University of London, 1 Myddelton Street, London, EC1R 1UW UK; 2 Nursing Midwifery and Allied Health Professions, University of Stirling, Stirling, Scotland FK9 4LA UK

**Keywords:** Pregnancy, Childbirth, Birthplace, Metasynthesis, Decision making, informed choice, Qualitative, Choice

## Abstract

**Background:**

The purpose of this systematic review (PROSPERO Ref: CRD42017053264) was to describe and interpret the qualitative research on parent’s decision-making and informed choice about their pregnancy and birth care. Given the growing evidence on the benefits of different models of maternity care and the prominence of informed choice in health policy, the review aimed to shed light on the research to date and what the findings indicate.

**Methods:**

a systematic search and screening of qualitative research concerning parents’ decision-making and informed choice experiences about pregnancy and birth care was conducted using PRISMA guidelines. A meta-synthesis approach was taken for the extraction and analysis of data and generation of the findings. Studies from 1990s onwards were included to reflect an era of policies promoting choice in maternity care in high-income countries.

**Results:**

Thirty-seven original studies were included in the review. A multi-dimensional conceptual framework was developed, consisting of three analytical themes (‘Uncertainty’, ‘Bodily autonomy and integrity’ and ‘Performing good motherhood’) and three inter-linking actions (‘Information gathering,’ ‘Aligning with a birth philosophy,’ and ‘Balancing aspects of a choice’).

**Conclusions:**

Despite the increasing research on decision-making, informed choice is not often a primary research aim, and its development in literature published since the 1990s was difficult to ascertain. The meta-synthesis suggests that decision-making is a dynamic and temporal process, in that it is made within a defined period and invokes both the past, whether this is personal, familial, social or historical, and the future. Our findings also highlighted the importance of embodiment in maternal health experiences, particularly when it comes to decision-making about care. Policymakers and practitioners alike should examine critically current choice frameworks to ascertain whether they truly allow for flexibility in decision-making. Health systems should embrace more fluid, personalised models of care to augment service users’ decision-making agency.

## Background

‘Choice’ has increasingly become a fundamental aspect of Western public health policy and practice, since the late twentieth century. In United Kingdom’s (UK) National Health Service (NHS), for instance, informed choice is considered an ethical principle that guides ‘patient-centred care’. For maternity care, it is a buzzword that has engendered a growing body of literature around how to best facilitate decision-making and achieve informed choice among parents. However, promoting more service user autonomy and care experiences that are aligned with personal values can lead to complex trade-offs, as choices are based on more sophisticated, expanding services with less reliance on clinician’s intuitive judgment [1]. The issue remains that, while maternity care professionals believe they are offering choice, in reality, women still have a limited role in decision-making and do not feel their care is presented as a choice. [2, 3] Some researchers have argued that informed choice, in particular, is, “at best, illusory”. [4, 5] Presently, there are few systemic reviews related to it, or to women’s experiences of decision-making about pregnancy and birth care. We sought to address this gap and assess the qualitative research that has been undertaken to date.

### Concepts of decision-making

Long-standing conceptualisations of decision-making and informed choice often assume that the decider uses a rational process to weigh up and decide between all available options [6]. This ‘rational process’ is connected to the tenets of classical decision theory, a concept that stretches across several disciplines, statistics, economics, neuroscience, physiology and psychology, and has been developed and discussed since the 1950s. [7] Classical decision theory is a collection of models of uncertainty, risk and utility that “prescribe the optimal choice of an option from an array of options, where optimality is defined by the underlying models and the choice is dictated by an explicit rule”.[[8] p21].

The quality of human decision-making, in actuality, is frequently evaluated using the standards of classical decision theory; however, this has not rectified the persistent issue that human behaviour does not necessarily conform to these standards and cannot be adequately described by them.^8^ Moreover, the long-held, assumed prescriptive and normative functions of the theory means that contemporary models of decision-making continue to inhabit these roles. The well-established health care model of ‘shared decision-making’ (SDM), “an approach where clinicians and patients share the best available evidence when faced with the task of making decisions, and where patients are supported to consider options, to achieve informed preferences” [[9] p1361], is framed as an idealised process that flows from smooth interactions between health care professionals (HCPs) and service users.

The SDM approach aims for HCPs to facilitate service users’ movement from ‘initial preferences’ to ‘informed preferences’. Although the model steps away from clinicians making decisions outright for people, ‘preferences’ implies that service users are still not in full control of the ultimate care decisions or choice outcomes. The authors of the SDM model do acknowledge that it “is a simplification of a complex, dynamic process”, and as an educational tool for clinicians, it may prove effective; however, the extent to which it gives services users more power in the decision-making process is not well established, nor is there evidence of any association between SDM and service user health outcomes [10, 11].

### Emerging models of maternity care

Outlining the assumptions underpinning decision-making and informed choice is important given the growing evidence of the benefits of different models of and options for maternity care in Western settings, and the prevailing notion of reproductive choice as a human right. Research demonstrating the positive clinical and social outcomes of midwifery-led continuity of care models [12], non-hospital birth settings [13–15] and reduced medical interventions [16] means there is an evidence base to underpin and strengthen the desirability of providing options. However, slow or stagnant uptake of new options among service users can be confounding, meaning policymakers and HCPs often rely on experiences of decision-making and choice to inform the best way to go about facilitating it. With the amalgamation of evidence concerning the clinical outcomes of different care options, it should follow that we also bring together the findings about women’s experiences of choosing these options. However, to date, there are few systematic reviews of qualitative research on women’s experiences of decision-making and informed choice. Previous reviews have focused on decision-making in regards to antenatal screening [17] and delayed childbearing [18], what women value during childbirth [19] and how informal information sources influence birth decisions. [20]

In this review, against the changing maternity service landscape, we ask: what are women’s experiences of decision-making about pregnancy and birth care, and how is informed choice being addressed in this research to date? There are many definitions of informed choice; it is generally seen as a choice that is based on availability of relevant and balanced information. For the purposes of this review, we did not seek to provide a working definition of informed choice, as this forms part of our focus of enquiry. The review instead sought to include all studies with a stated focus on the issue, each of which could potentially define informed choice in a different way.

## Methods

As the review encompassed published qualitative data, we employed a meta-synthesis approach to explore the relevant literature, which is anchored around “bringing together and breaking down of findings, examining them, discovering essential features and, in some way, combining phenomena into a transformed whole”.[[21] p314] This new interpretation of, or ‘going beyond’ [22] research is one of the key aspects that sets a meta-synthesis apart from a meta-analysis, which aggregates findings to establish ‘truths’. The search and screening were conducted using the Preferred Reporting Items for Systematic Reviews and Meta-Analyses (PRISMA) guidelines, an evidence-based framework used for reporting in systematic reviews [23, 24], and was registered on the PROSPERO (Ref: CRD42017053264) database of protocols for systematic reviews.

### Reflexivity

The review team was composed of four researchers who specialise in maternal and child health, maternity care and services and midwifery, and who conduct qualitative research on parents’ experience of maternity and birth care, primarily in the UK and Brazil. The first (CY) and second (CM) reviewers are medical anthropologists, and the third (HC) and fourth (NL) reviewers have backgrounds in midwifery, meaning we approached this review and meta-synthesis with an interdisciplinary lens.

### Systematic search and screening

The initial search for articles was conducted on EBSCO (Academic Search Complete, CINAHL, Medline, SocIndex, PsycARTICLES), OVID (Embase, Global Health, Maternity and Infant Health Care) and Web of Science in January 2017. The grey literature was searched using OpenGrey and EThOs. A second search, which limited the results to articles published between 2017 and 2019, was run in August 2019 to check for new literature. Study content was not limited to a specific geographic region; however, articles had to be published in English. The searches focused on pregnancy and childbirth and did not include family planning, infertility, abortion or postnatal care as the focus was on choices about pregnancy and birth *care*. The search was filtered for studies employing qualitative or mixed methods research designs and analyses, which was key for the data extraction and synthesis process. Quantitative studies and quantitative findings from mixed methods studies, RCTs and open-ended questions from survey studies were excluded. The search strategy included Boolean phrases of “AND” and “OR”, and terms were generated using MESH headings, database thesaurus and free text. An example of the search strategy that was used on EBSCO is detailed in Table 1.

**Table 1:** An example of the search strategy used on EBSCO

Informed choice	1. (MH “Decision Making, Patient”) 2. AB “Informed choice” OR AB “informed decision making” OR AB “decision making 3. **1 OR 2**
Maternity care	4. (MH “Maternal-Child Care”) OR (MH “Intrapartum Care”) OR (MH “Perinatal Care”) 5. (MH “Childbirth”) OR (MH “Pregnancy”) 6. AB “maternal health” OR AB “maternity care” OR AB childbirth OR AB birth OR AB labour OR AB “intrapartum care” OR AB “obstetric care” OR AB pregnancy 7. **4 OR 5 OR 6**
Women’s experiences	8. (MH “Women”) 9. (MH “Mothers”) or (MH “Expectant Mothers”) 10. AB women N5 experiences OR AB women N5 perceptions OR AB women N5 views OR AB women N5 opinions OR AB women N5 attitudes OR AB women N5 perspectives OR AB women N5 accounts OR AB women N5 narrative OR AB women N5 story OR AB women N5 stories 11. AB mothers N5 experiences OR AB mothers N5 perceptions OR AB mothers N5 views OR AB mothers N5 opinions OR AB mothers N5 attitudes OR AB mothers N5 perspectives OR AB mothers N5 accounts OR AB mothers N5 narrative OR AB mothers N5 story OR AB mothers N5 stories 12. **8 OR 9 OR 10 OR 11**
Qualitative studies	13. (MH “Qualitative Studies”) 14. AB “qualitative research” OR AB “qualitative methods” OR AB “mixed methods” OR AB interview OR AB “focus groups” OR AB diary OR AB diaries OR Ab ethnography 15. **13 OR 14**
Full search	16. **3 AND 7 AND 12 AND 15**

Filter: Humans, from 1990, English language

Articles were screened for fit with the inclusion criteria independently by two reviewers (CY and CM), first by title, then by abstract and following this by full text. In cases of uncertainty or disagreement, the third (HC) and fourth (NL) reviewers’ views were sought. Although potentially relevant to a wider discussion, to maintain the focus of the review on decision-making and choice in the context of more standard care pathways. Therefore, we excluded studies with a focus on a specific risk factor or in relation to a specific intervention. Our search produced a good number of papers about both VBAC and planned caesarean section. We felt that these had more of a specific focus and decision-making contexts that warranted separate reviews in order to explore the themes and unpack the complexities related to each. Table 2 defines the inclusion and exclusion criteria that were used during screening.

**Table 2:** The inclusion and exclusion criteria used during the screening of search results

	Inclusion	Exclusion
Participants	Women who are primiparous or multiparous, at any gestational term, of any mode of birth, or experienced either a facility-based or non-facility-based birth	Women who have not brought a viable pregnancy to full term, birth partners who are not fathers, health care professionals
Intervention	Investigating informed choice in maternal health, specifically what influences it and women’s experiences of making decisions about their maternity care	VBAC, Specific focus on maternity service use and access, reproductive choices, infertility treatment, HIV/AIDS in pregnancy, health behaviours in pregnancy, foetal screening, or decision-making about specific risks or complications, VBAC, planned caesarean section, post-natal care and practices, clinical or technical quality of care only, women’s experiences described by others (e.g. health professionals)
Outcomes	Any	N/A
Study design	Primary qualitative studies, including, but not limited to, ethnography, phenomenology, grounded theory and feminist research. Qualitative components of mixed methods or experimental studies	Quantitative studies, RCTs, quantitative findings from mixed methods designs Open-ended questions in survey studies
Study focus	Exploration of women’s informed decision-making about their maternity care and health, specifically what influences choice and women’s experiences of this decision-making process	Main focus is not on exploration or women’s experiences of informed decision-making in a maternal health context
Setting	High-income countries, middle-income countries with comparable health care system and socio-cultural background to the United Kingdom	Low-income countries, mid-income countries where health system and socio-cultural background is not comparable to that of the United Kingdom
Time period	1990–2017	Before 1990
Language	English	All other languages
Publication type	Peer-reviewed articles, theses, research reports	Reviews, opinion articles, policy documents

### Quality appraisal

Three reviewers (CY, CM and HC) independently carried out a quality assessment of the included studies using a tool [25] for qualitative research that was adapted from Walsh and Downe. [26] Initially, we intended to use CASP [27] for the qualitative research appraisal; however, this tool, though popular, was found to be less sensitive to validity than other critical appraisal tools for qualitative research. [28] We amended the tool by Rocca-Ihenacho during our quality appraisal, decreasing the number of items on the checklist and adding in a more nuanced scoring system. The resulting tool (Table 3) uses 33 items to appraise research on the basis of scope and purpose, methodology, research design, sampling strategy, data collection and analysis, interpretation of data, discussion of results, reflexivity and ethical considerations. Studies were scored from 0 to 2 for each item to denote the quality of each item and were given an overall quality rating (0–22 = low; 23–44 = moderate; 45–66 = high). In the few cases of uncertainty or disparity, the reviews outlined their rationale for scoring and came to an agreement based on this discussion.

**Table 3:** Adapted tool by Rocca-Ihenacho used for the quality appraisal

Stages	Specific elements	Essential criteria	Score
Rational	Scope and purpose	1. Contextualization with literature	
		2. Aims	
		3. Research question or objectives stated	
Design	Methodology	4. Rational for using qualitative design	
		5. Description of theoretical background	
		6. Description of methodology	
		7. Methodology appropriate for research question or objectives	
	Methods	8. Description of methods	
		9. Methods appropriate for research question or objectives	
	Sampling strategy	10. Rational for sampling strategy explained	
		11. Selection criteria described	
		12. Thickness of description likely to be achieved from sampling	
	Data collection	13. Description of data collection	
		14. Data collection strategy appropriate to capture complexity of events and highlight context	
	Analysis	15. Analytical approach explicit	
		16. Analytical approach appropriate for methodology	
		17. Analysis grounded in the data	
		18. Evidence of participants’ involvement in analysis	
		19. Saturation addressed	
Interpretation	Clear audit trail given	20. Demonstration of thorough interpretive pathway and ‘decision trail’	
	Description of context	21. Description of social, physical and interpersonal contexts of data collection	
	Interpretation grounded in the data	22. Extensive use of field notes entries/verbatim interview quotes in discussion of findings	
		23. Provides new insights and increases understanding	
Discussion	Contextualization with literature	24. Findings compared and contrasted with other literature	
	Relevance and transferability	25. Interpretation interwoven with existing theories and other relevant literature drawn from similar settings	
		26. Discussion of how explanatory proposition/emergent theory may fit other contexts	
		27. Limitations and weaknesses of study clearly outlined	
		28. Significance for current policy and practice outlined	
		29. Outlines further directions for investigation	
Reflexivity	Researcher reflexivity demonstrated	30. Discussion of relationship between research and participants during fieldwork	
		31. Discussion about how issues/complications met were dealt with	
Ethical dimensions	Ethical committee approval	32. Evidence of ethical approval and following ethical procedures	
	Sensitivity to ethical concerns	33. Documentation of how autonomy, consent, confidentiality, anonymity were managed	
		**Total**	

### Data extraction and synthesis

The first reviewer (CY) extracted data from the selected studies to assess quality and to synthesise reported results with supervision from the second reviewer (CM). No discrepancies were identified during this process. The extracted qualitative data was coded line-by-line in NVivo to enable the translations of concepts from one study to another and to build a qualitative synthesis, following Thomas and Harden’s [22] methodology. This approach enables the synthesis to ‘go beyond’ in order to identify key concepts in the studies and translate them into one another. The process of translation allows the recognition of similar concepts used within studies, even if they are not explicitly stated as such. The theories associated with these concepts are extracted so that a line of argument can be developed and concordant concepts can be put together, bringing fresh interpretations.

### Search results

**Fig. 1:**
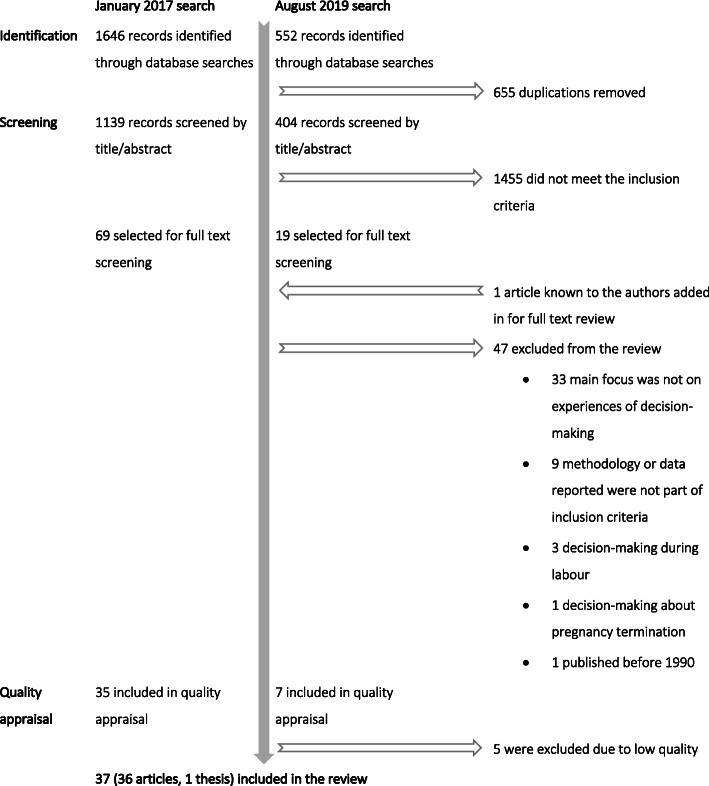
Flow chart of results of the record identification, screening and quality appraisal for the review

The searches identified 2198 records, and after screening both titles and abstracts, 88 were selected for full text review. One article known to the authors was added, bringing the total to 89 records. Of these, 47 were excluded for a number of reasons. The most common was because the study focused on an aspect of women’s experiences outside of decision-making about pregnancy and birth care, or there was little to no inclusion of decision-making or informed choice in the study aims or findings. From the remaining records, five were removed because they were scored as low-quality during the quality appraisal, leaving 37 records that were included in the meta-synthesis. Twenty-five were rated moderate quality, and 12 were of high quality. A majority of the studies included in the final review were peer-reviewed journal articles. One PhD dissertation [29] was included; however, the findings extracted from the text were done with care so that the extended format did not dominate the analysis or the emergent themes. The search results can be viewed in Fig. 1.

## Results

### Descriptive findings

All of the studies reported in the review were conducted in nine countries, the UK (17), the US (7), Canada (5), Australia (4), New Zealand (5), Finland (2), Denmark (1), the Netherlands (1) and Spain (1). Several of the articles included in the review reported on studies that were conducted in multiple countries, most notably Lagan et al. [30], which was undertaken in five countries. The range of countries included is small because the inclusion criteria was limited to research published in English, and to studies conducted in countries with comparable medical systems to the UK. Table 4 provides a summary overview of the included studies with their corresponding numbers.

**Table 4:** Summary of included studies

Reference number	Study	Country	Study aims	Participants, setting	Study design, data collection, analysis	Quality
[31]	Andrews, 2004	United Kingdom	To explore women’s experiences of home birth	• 8 women who had planned a home birth in the six months before the study • South Wales	• Qualitative interview study • Semi-structured interviews using open-ended questions • Thematic analysis using a phenomenological approach	M
[32]	Bedwell, et al., 2011	United Kingdom	To explore expectant fathers’ views of birth setting	• 37 expectant fathers, 19 of which were interviewed • North West of England	• Qualitative interview study using interpretive approach • Semi-structured interviews in women’s 34th week of pregnancy • Thematic analysis	M
[33]	Bernhard, et al., 2014	United States of America	To explore why women choose home birth and their perceptions of their birth experiences	• 20 women who had a hospital birth and subsequently chose a home birth • Not stated	• Qualitative description study • Five focus groups conducted with participants • Qualitative content analysis	M
[34]	Catling, et al., 2014	Australia	To explore what influences women who chose a publicly-funded home birth in one state in Australia	• 17 women who chose a publicly-funded home birth • Suburbs of Sydney, Australia	• Qualitative interview study using a constructivist grounded theory approach • Semi-structured interviews were conducted with participants • Analysis method not stated	M
[35]	Catling-Paull, et al., 2010	Australia	To explore the reasons why multiparous women feel confident to have a home birth within a publicly-funded model of care in Australia.	• 10 multiparous women who chose to have a home birth within a publicly funded care model • Not stated	• Qualitative interview study • Postnatal semi-structured interviews using open-ended questions • Thematic analysis	M
[36]	Cheung, 2002	United Kingdom	To provide insights into how women’s birth experiences can be improved	• 10 Scottish and 10 Chinese pregnant women • Scotland	• Qualitative study • Four semi-structured interviews (antenatal and postnatal) with the 20 expectant mothers • Thematic analysis filtered through participant observation	H
[37]	Coxon, et al., 2014	United Kingdom	To examine the extent to which approaches to risk can enhance understandings about birthplace decision-making	• 41 women who access maternity services • Two inner-city area and one semi-rural area in the United Kingdom	• Longitudinal narrative study • Three interviews with all participants • Thematic narrative analysis	H
[38]	Coxon, et al., 2015	United Kingdom	To explore the influence of pregnancy and birth experiences on women’s current and future place of birth decisions	• 41 women who access maternity services • Two inner-city area and one semi-rural area in the United Kingdom	• Prospective, longitudinal narrative study • Three interviews with all participants • Thematic and structural narrative analysis	M
[39]	Dahlen, et al., 2008	Australia	To explore the experiences of first-time mothers who gave birth either at home or in the hospital in Australia	• 19 first-time mothers • Not stated	• Qualitative study using a grounded theory approach • In-depth interviews were conducted with participants 6 weeks after they had given birth • Grounded theory analysis procedure using open, axial and selective coding	M
[40]	DiFilippo, 2015	Canada	To examine women’s learning when choosing to give birth at home with a midwife using a critical feminist approach	• Seven women who planned a midwife-attended home birth in the two years prior • Ontario, Canada	• Qualitative study • Semi-structured interviews • Close textual analysis in order to identify themes	M
[41]	Feeley & Thomson, 2016	United Kingdom	To explore what influences women’s decisions to free birth within the United Kingdom	• 10 women planning to freebirth • Not stated	• Qualitative study using an interpretive phenomenological approach • Narratives and in-depth interviews were conducted with participants • Analysis guide by Heidegger and Gadamer’s interpretive phenomenological concepts	H
[42]	Grigg, et al., 2014	New Zealand	To explore and report what influences women’s decision-making about place of birth in New Zealand	• 37 women from the larger study cohort (*n* = 702) • Christchurch, New Zealand	• Mixed methods study • Qualitative data generated from focus groups conducted in Christchurch • Inductive content analysis	M
[43]	Grigg, et al., 2015	New Zealand	To explore women’s birthplace decision-making; to identify what enables a women to plan giving birth in freestanding midwifery-led unit rather than an obstetric unit	• 37 women from the larger study cohort (n = 702) • Christchurch, New Zealand	• Mixed methods prospective cohort study • Eight focus groups were conducted • Thematic analysis	M
[44]	Happel-Parkins & Azim, 2016	United States of America	To explore and contextualise the experiences of first time mothers who planned a natural birth (e.g. No medical intervention)	• 6 first-time mothers • Midsouthern region of the United States	• Narrative inquiry study • Semi-structured, life-story interviews were conducted • Thematic analysis	H
[45]	Jimenez, et al., 2010	Canada	To explore women’s birth experience in the context of the changes that have occurred in perinatal care since the 1970s; to examine how acquired information and knowledge about birth and pregnancy influence women’s birth experiences	• 36 pregnant women (26 from Montreal, 10 from Vancouver) • Montreal and Vancouver, Canada	• Qualitative interview study • Two semi-structured interviews at 4–6 weeks before birth and 6–8 weeks after birth were conducted with participants • Thematic analysis	M
[30]	Lagan, et al., 2011	Australia, Canada, New Zealand, United Kingdom and United States of America	To build on previous quantitative studies on women’s internet usage for pregnancy-related information; to explore women’s experiences and perceptions of using the Internet for pregnancy-related information and its influence on their decision-making	• 92 women from five countries • Online, specific regions not stated	• Global study drawing on interpretative qualitative traditions • 13 asynchronous online focus groups • Inductive thematic analysis using Ritchie and Spencer’s framework	M
[46]	Lally, et al., 2014	United Kingdom	To explore how women can be better supported when preparing for and making decisions about pain management during pregnancy and labour	• 32 pregnant women • North east region of England	• Qualitative interview study • Semi-structured interviews conducted when women were 28–36 weeks pregnant and six weeks after they gave birth • Thematic analysis	M
[47]	Lee, et al., 2016	United Kingdom	To examine the decisions about place of birth among women with high-risk pregnancies, who were planning either home or hospital births	• 26 women with high-risk pregnancies, who were at least 32 weeks gestation • Not stated	• Qualitative interview study • Semi-structured interviews • Inductive thematic analysis	H
[48]	Levy, 1999	United Kingdom	To explore the processes involved when women make informed choices during pregnancy	• 17 pregnant women receiving care in different maternity settings • East Midlands, England	• Qualitative study using grounded theory approach • Observation during booking appointments of 12 women, with follow-up interviews • Five interviews and one observation with women who were 32–38 weeks pregnant to aid theory construction • Thematic analysis using a grounded theory approach	M
[49]	Lothian, 2013	United States of America	To explore women’s experiences of planning, preparing for and having a home birth in the United States	• 13 pregnant women who were planning a home birth in the United States • Not stated	• Ethnography • Informal interviews and participant observation during participants’ pregnancies and after the births of their babies • Thematic analysis following Lincoln and Guba’s guidelines	M
[29]	Madi, 2001	United Kingdom	To explore pregnant women’s decisions about place of birth and what influences their preferences	• 33 pregnant women (20 planning a hospital birth, 13 planning a home birth • Not stated	• Qualitative interview study • Semi-structured interviews were conducted with all participants • Thematic analysis	H
[50]	Malacrida, 2015	Canada	To examine how women learn about birth and make choices using a critical feminist perspective	• 40 expectant and new mothers • Red Deer and Lethbridge, Alberta, Canada	• Qualitative interview study • Qualitative, semi-structured interviews • Thematic analysis	M
[51]	Mander & Melender, 2009	Scotland, Finland and New Zealand	To examine if choices and decision-making could be enhance for pregnant women in Scotland; to inform the organisation of Scottish maternity services	• 4 women who use the maternity services • Not stated	• Qualitative study using a hermeneutic phenomenological approach • In-depth, semi-structured conversations • Modification of Colaizzi’s analysis procedure for phenomenological research	H
[52]	Miller & Shriver, 2012	United States of America	To explore women’s perceptions and decision-making regarding birth in an American context	• 135 women who chose either a home birth with a midwife, an unassisted home birth or a hospital birth • Southeastern region of the United States	• Three phase qualitative and ethnographic study • Phase one involved interviews with 60 women, phase two involved in-depth interviews with 21 interviews supplemented by a dataset of 127 birth stories and phase three was an ethnography of birth in an American obstetric unit • Line-by-line coding analysis of interview transcripts and database birth stories • Contextual analysis of field notes	H
[53]	Murray-Davis, et al., 2012	Canada	To increase understanding of why women decide to have a home birth; to describe what influences women’s decision to plan a home birth	• 34 women who were either pregnant and planning a home birth or who had planned a home birth in the last two years • Ontario and British Columbia, Canada	• Qualitative interview study using a grounded theory approach • Semi-structured interviews • Thematic analysis	M
[54]	Pitchforth, et al., 2009	United Kingdom	To explore women’s experiences of choice of birthplace in remote and rural area where different models of maternity services	• 70 women who had given birth in the prior 7 years • Remote and rural areas of North Scotland	• Qualitative focus group study • 12 focus groups at eight study sites • Analysis using an inductive thematic approach	H
[55]	Regan, et al., 2013	United States of America	To examine the factors that influence women’s decisions about birth and how this affects caesarean section use	• 49 first-time mothers between the ages of 21 to 36, who were in their 28-36th week of pregnancy • Not stated	• Mixed methods study • Focus groups and structured postpartum interviews with all participants • Analysis using the Consensual Qualitative Research method	H
[2]	Stapleton, et al., 2002	United Kingdom	To examine how evidence-based leaflets about informed choice are used in maternity services	• Pregnant women were recruited from maternity units • Cohort size not reported • Not stated	• Randomised controlled trial using qualitative methods • 886 non-participant observations of antenatal consultations and 173 in-depth, semi-structured interviews (85 antenatal, 78 postnatal) • Not stated	M
[56]	Song, et al., 2012	United States of America	To explore how white women use the Internet during their experiences of conception, pregnancy and birth To examine the extent to which this usage aids in making meaningful choices and shapes their patient identities	• 32 women who identified as Caucasian • Southeastern region of the United States	• Qualitative interview study using grounded theory approach • Interviews were conducted with each of the 32 participants • Thematic analysis using the inductive approach of grounded theory	M
[57]	Viisainen, 2001	Finland	To explore how cultural models of birth and current practical choices influence parents’ understanding of home birth; to examine women’s reasons for and experience of planning a home birth	• 21 women and 12 men who had planned to give birth at home within the prior three years • Not stated	• Qualitative interview study • Unstructured interviews with open-ended questions were conducted with 12 couples and nine mothers • Narrative structuring used for analysis	M
[58]^a^	Borrelli, et al., 2017	United Kingdom	To explore what influences first-time pregnant women’s choice of birthplace; to examine women’s expectations of the midwife’s role in different birthplaces and what they perceive as safe in regards to different settings	• 14 women in good general health expecting their first baby, with a low-risk pregnancy and anticipating a straightforward birth • England	• Qualitative interview study using Straussian grounded theory methodology • Semi-structured interviews were conducted with 14 women in-person • Strauss and Corbin analytical grounded theory approach	H
[59]^a^	Hinton, et al., 2018	United Kingdom	To examine what factors are important to women when making a choice between different birthplaces; which attributes of maternity services women value; what services are needed for NHS trusts to provide women a realistic choice of home birth; what are the effects of travel time and distance on women’s choices; how women access and evaluate information about birthplace options	• 69 women in their last trimester of pregnancy • Online (England), London	• Qualitative focus group study • Seven focus groups conducted online on a bespoke web portal, one conducted face-to-face • Analysis employing a combination of a thematic framework and the ‘One Sheet of Paper’ method	M
[60]^a^	Hollander, et al., 2017	The Netherlands	To explore the motivations of Dutch women who have chosen to give birth ‘outside the system’ (e.g. against medical advice and/or guideline/protocol)	• 28 women who had chosen to ‘birth outside of the system’ for one or more of their pregnancies • Not stated	• An exploratory qualitative research design with a constructivist approach and a grounded theory method • In-depth interviews with 28 women, one focus group • Thematic analysis using open, axial and selective coding	H
[61]^a^	Leon-Larios, et al., 2019	Spain	To explore the perceptions, beliefs and attitudes of women who opted for a home birth in Andalusia, Spain	• 13 women who had chosen a home birth in the past five years • Andalusia	• Qualitative interview study with phenomenological approach • Face-to-face semi structured interviews • Thematic analysis using a phenomenological approach	M
[62]^a^	Naylor Smith, et al., 2018	United Kingdom	To identify the factors that influence women’s choice of place of birth, and to explore their views of home birth	• 28 low-risk, multiparous women • Large, ethically diverse city in the UK	• Qualitative focus group study with interpretative approach • Five focus groups with 28 women in routine mother and baby groups • Thematic analysis using the Framework Method	M
[63]^a^	Patterson, et al., 2017	New Zealand	To explore retrospectively the choice of birth place decisions and the labour and birth experiences of women living in remotely zoned, rural areas of New Zealand	• 13 women living in a remote rural area who had given birth in the past 18 months • Rural Otago and South Isand	• Qualitative interview study using a pragmatic interpretative approach • Semi-structured interviews with participants, field notes • Thematic and content analysis using Aronson’s pragmatic approach	M
[64]^a^	Tayyari Dehbarez, et al., 2018	Denmark	To investigate pregnant women’s decision making in relation to their choice of birthing hospital and, in particular, their priorities regarding hospital characteristics	• 13 low-risk pregnant women in their first trimester who had attended their first antenatal consultation and had been presented with a choice of hospitals • Central Denmark Region	• Qualitative interview study • Semi-structured interviews, with follow-up interviews conducted over the phone • Thematic analysis	M

^a^ Denotes results from the second search conducted in August 2019

While all of the research broadly focused on decision-making and informed choice, there was a range of study topics, methods, sample sizes and analytical frameworks used to investigate the aims. A majority of the research centred on place of birth. [29, 31–35, 37–43, 47, 49, 53, 54, 57–64] Of these, 10 focused exclusively on home birth [31, 33–35, 40, 49, 53, 57, 60, 61] and one on free birth. [41] The next most common study topics were decisions about birth [50–52, 55] and influence of information sources [30, 45, 56]. Only two studies [2, 48] were concerned with informed choice as a primary study aim, and, finally, three studies explored birth experiences [36], natural birth [44] and pain management. [46] Overall, place of birth research was disproportionally represented in the studies identified.

There was a variety of study designs employed in the research under review. Most [29, 31–36, 38–40, 44–47, 50, 51, 53, 56–58, 60, 61, 63, 64] exclusively used interviews to collect data. Seven studies [30, 33, 42, 43, 54, 59, 62] gathered data using focus groups, and the rest used a combination of qualitative methods, usually interviews with either participant observation [49, 52], non-participant observation [2, 48], narratives [41] or focus groups. [55] Sample sizes ranged from four to 135 participants, although one study [2] did not report their sample size. The average study size among the records reviewed was approximately 32. Most of the studies [29–32, 35–38, 43–47, 49, 50, 53, 54, 57, 59, 60, 62–64] used thematic analysis to generate results, while the remaining studies employed either theory-guided analysis [39, 41, 48, 51, 56, 57, 61], qualitative content analysis [33, 42], close textual analysis [40, 52] or consensual qualitative research analysis [55]. Two papers did not state their analysis methods. [2, 34]

The review included only two papers [32, 57] that incorporated partners’ experiences of choosing place of birth, and, of these two, one [32] focused exclusively on fathers. There are also few ethnographic studies [49, 52] of decision-making and informed choice in maternal health: most of the articles we reviewed employed either grounded theory [34, 39, 48, 53, 56, 58, 60], phenomenological [31, 41, 51, 61] or narrative [37, 38, 44] approaches as study designs. In general, the reporting of theoretical and methodological aspects of research was not as frequent as expected; for example, only 18 of the 37 studies reviewed stated their methodological frameworks. Because decision-making and informed choices are not straightforward processes and are enmeshed in wider socio-cultural relationships, it is important to situate the qualitative methodology within social theory. Moreover, we found little anthropological or sociological research on decision-making in maternal health, despite a rich body of work already established on reproductive and maternity experiences, both in the US and UK (see Davis-Floyd and Kitzinger).

### Synthesis findings

**Fig. 2:**
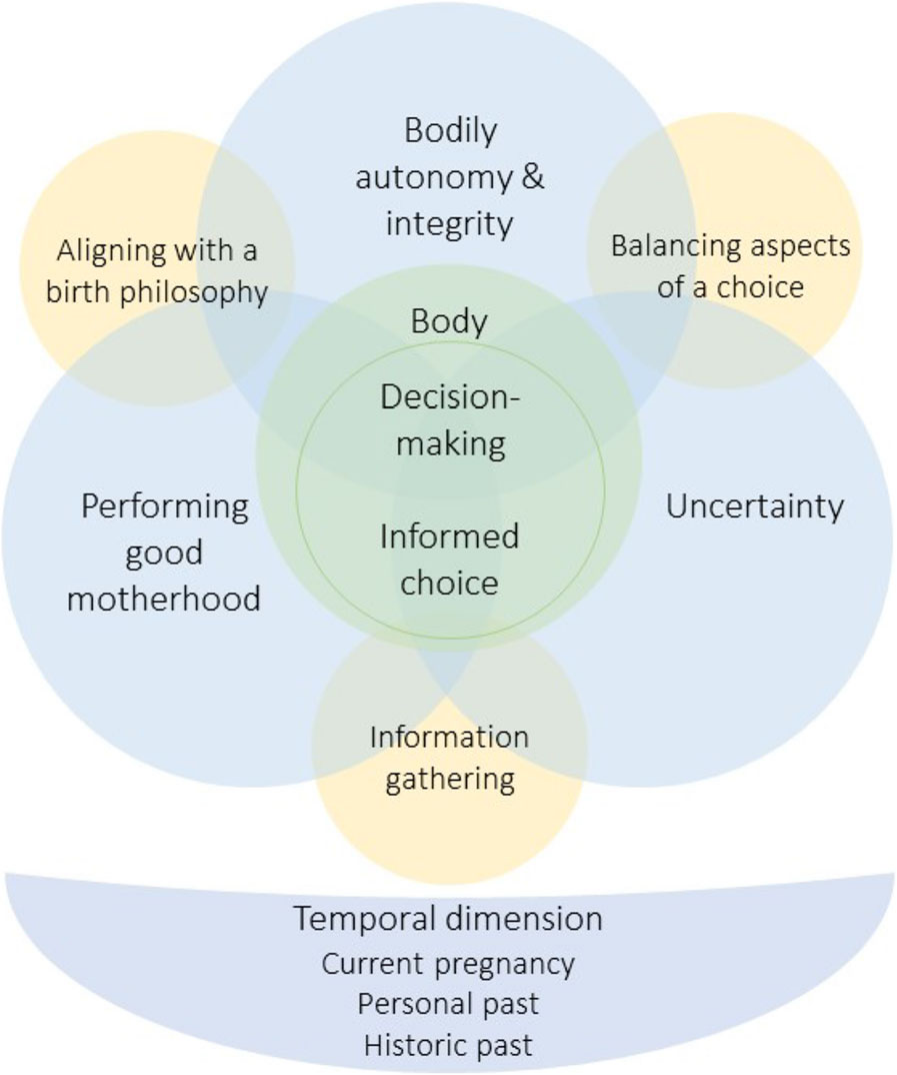
Conceptual map of the synthesis findings, showing interaction of inter-linking actions, themes and temporal dimensions

Informed by women and their partners’ accounts of their decision-making experiences and the descriptive level of analysis, three overarching analytical themes emerged – ‘Uncertainty’, ‘Bodily autonomy and integrity’ and ‘Performing good motherhood’. These core themes are interwoven and overlapping as they reinforce and feed back into each other, and certain aspects of one can be confluent with those of another. There are three inter-linking actions – ‘Information gathering’, ‘Aligning with a birth philosophy’ and ‘Balancing aspects of a choice’ – as decision-making is an active albeit abstract process. The themes and inter-linking actions are framed by a ‘Temporal dimension’ that provides multidimensional depth to the process of decision-making and concept of informed choice. Figure 2 provides a visual conceptual map of the synthesis findings. All quotes in the following discussion are from the texts and participants of the original studies under review.

#### Inter-linking actions

##### Information gathering

This action illustrates the multiplicity of information-seeking and what women specifically gain from different sources. While women may seek information from sources outside of the medical sphere, this does not mean that they do not rely on information from HCPs, who are still seen as valuable providers, particularly as active rather than static sources:

“I have had the pamphlets that the midwife gave me but I feel that if there were anything I really wanted to know then I would ring the midwife--my own midwife at the surgery … it’s just that you can have a conversation about it...in a book it’s just written there, whereas you can discuss it when you are with the midwife.”[[48] p114].

Trust is a crucial strategy for navigating convoluted decisions, particularly trust in experts with the appropriate skills and knowledge. [65, 66] As the participant above illustrated, the pamphlets from her midwife only provided so much, it was rather informal conversations that were more valued. Though HCPs are seen as trustworthy sources, women consistently reported looking for information, particularly for more detailed knowledge, outside of their appointments and beyond the ‘formal’ sources to family, friends and the Internet. These may provide a more comprehensive picture of care or specifics about different options, or be used to validate or confirm what HCPs have said:

Many wrote that they used the Internet to clarify information received from other sources—health professionals, family friends, or literature, such as pregnancy books and leaflets: “For me it was more to confirm what a doctor had said. Silly, I know, but I just wanted to understand it some more, which it helped me to do.”[[30] p342].

Other sources of information can also be personal experiences, and care choices may stem from this internal history, particularly when women come to trust themselves and their bodies, which is common among those who chose to home or free birth:

“I knew that with my second, I was searching for a greater depth to the experience, something more intuitive as I had come to trust myself more than I had previously, not just through experience, but through research that supported my beliefs in understanding the science behind mammalian instinct, physiological birth and the huge value of the hormonal and emotional process.”[[41] p7].

Information is a way to counteract uncertainty, whether it pertains to fear or safety, while simultaneously functioning as a space in which women perform good motherhood, gathering all the resources possible to demonstrate their knowledgeability and responsibility as a mother:

“I had a lot of fear around pain and what I considered to be this maiming experience of birth. I didn’t read anything for 9 months except birthing books, I swear. I mean, I just studied it. I knew it so well that the birthing classes suggested that I become a teacher. By the time I was in preparation for her birth, I had a lot of very, very positive expectations around the birth.”[[39] p27].

The woman above has not only used intensive research to overcome her uncertainty and dread but has also been socially bestowed as an expert who is so knowledgeable that she can teach a birthing class herself. Information gathering can be used to shift an emotional outlook, whether it be for better or worse, relieving or inducing uncertainty, and it can also function as a strategy to forge or strengthen trust in sources of knowledge and in the self, by confirming what a HCP has advised or empowering the belief that birth can be positive, rather than “maiming”.

##### Balancing aspects of a choice

The next inter-linking action is concerned with looking at how women consider the different options available to them and, essentially, weigh the advantages and disadvantages of those they encounter. There are different everyday strategies that women use to navigate the many care decisions, including comparing information about an option or actively assessing and managing the uncertainty that is associated with their choice. ‘Balancing aspects of a choice’ focuses on the decision itself, how it is considered in relation to the self and the external world and how this shaped the ultimate choice outcome:

“I knew I had the information I was looking for generally after searching a number of sites and comparing a number of sources of information and when I felt satisfied I had learnt all that I could on a particular topic. I also liked to compare / discuss what I had found on the Internet with other sources, such as my midwife, obstetrician, GP, textbooks, etc.”[[30] p341].

“I have heard, of course, of the possibility of giving birth in a birthing center, but I thought it was not appropriate for me, because I think that it suits women who are calm and confident better. Me, I have more of an anxious nature. As I reflected during the course of my pregnancy, I came to the conclusion … I am too able to imagine all the problems that could occur. It makes me feel safe to be in a hospital.” [[50] p163].

Balancing involves understanding and managing uncertainty, developing “safety nets” in the event of a complication during labour and childbirth. This phenomenon is not a recent one. McClain [67, 68] found that women’s choices of birth services were based on balancing the risks and benefits of each option. Risk and benefit have thus remained strong vectors along which decisions are made against others; the central question being: what is the risk to my body and my baby?

“We just won’t let labour go on that long – if things aren’t progressing, we’ll ask for a caesarean section...I’ve done everything I can to be fit and healthy. I’ve done NCT [National Childbirth Trust] – very helpful apart from they’re a bit mad, and you have to take it with a pinch of salt. They are very pro- ‘active birth’ and anti-drug.”[[37] p61].

“So once I got past the anxiety issues, and making that final decision, and knowing that if I didn’t want to go ahead with it then I could always back out and still go to the hospital.”[[35] p125].

Women pursuing non-hospital births reported assessing and managing uncertainty regularly throughout their pregnancy, revealing the extent to which uncertainty is embodied and how bodily parameters orient decisions. Balancing the aspects of ‘alternative’ maternal care choices acquire legitimacy and potency as medical tests and clinical appointments confirm a ‘low-risk’ status, and optimal fitness, by these standards, is affirmed. So powerful are these biomedical definitions of health and risk that they determine pathways of care, even if it is generally outside its bounds and setting:

“I didn’t actually decide to have a homebirth until I had had my 28-week gestational diabetes test, because I had had a false positive with that, so then after I’d had my second test for that then I decided that yes, I wanted to go ahead and have a homebirth. I needed to have everything clear in my head that everything was going to be healthy for me to be able to have a homebirth.” [[35] p125].

Through this action, we can see how blended decision-making can be: it must encompass both physiological and social considerations, reflecting the dynamic nature of childbirth knowledge itself, which is comprised of biological processes and social praxis [69]. Balancing is an effective strategy for navigating these complex decisions and myriad information sources pertaining to them.

##### Aligning with a birth philosophy

In terms of decision-making, an important part of the process is aligning with a specific ‘birth philosophy’, or perspectives that inform the ways in which people understand the world and life in it, further shaping the information women access, the care opinions they ultimately choose and the ways they justify their choices. Birth philosophies, as they are presented in the literature reviewed, fall primarily into two camps; one views pregnancy and birth as ‘medical’ events, while the other sees each as ‘natural’ events. Many women’s perspectives reflect the medicalisation of childbirth in Western countries and as a result, they entrust their bodies, which can viewed as sites of danger, to medical care:

“I had a couple of people going ‘oh but it’s all just a natural process and it’s all good and you should be all fine’; well actually if you look around the world most of the women die in childbirth, that’s the riskiest thing women do; I wasn’t terribly impressed with that argument.”[[42] p10].

“I mean, it’s a medical thing. I’m not going to stay home if my appendix bursts either. I want the people who are trained and know what they’re doing to deliver my baby. Why wouldn’t you go to the hospital? That’s crazy!”[[52] p712].

Through this lens, placing one’s self anywhere but in a hospital and into medical care is “crazy”; however, women who see pregnancy as a natural event view ‘alternative’ options as opportunities to maintain their physical agency. These women often evoke the histories of women who have given birth outside of the hospital and the desire not to interfere with their bodies’ physiological processes. They do not conceive of their bodies as sites of danger, rather as those undergoing an organic event, and that care should indicate this:

“For me, pregnancy is a totally natural thing. If one is in good shape, in good health, there is no reason why one should go to the hospital to have access to medical help. Yes, things can go wrong, and this is why it reassured me to know that midwives are paired up with the hospital … But if things go well, I have no reason to ask for a doctor’s help.”[[45] p163].

“I think [TMH]- it’s a hospital, which if you are sick or if you’ve had an accident, that’s great, that’s exactly what you want; but I wasn’t sick, I was having a baby – it’s a perfectly natural process that millions of women all around the world have managed to do without nice shiny hospitals”[[43]p600].

The insinuation that giving birth outside of the hospital is right for – if not, limited to – women who are in “good shape” and “good health” reveals the overwhelming burden of risk discourse in maternal health. Despite this crossover between the ideological camps, there were indications in this review of emerging perspectives that allow each to dovetail, but increasingly underscore the importance of individualised choice as a way to display and promote appropriate motherhood and maternal care:

“For me a birth is natural when I can keep the child close to me and do what I feel right in the process, whether I will use technologies or I won’t. First and foremost the natural in birth means my choice and my decisions. If I feel that an intervention is necessary it is not against the idea of a natural birth, nor is the use of medicine.”[[45] p163].

Engaging with, forming and embracing a birth philosophy are all important steps along the decision-making process and contribute to a woman’s ability to make an informed choice about her care; however, it is essential that these ‘ideologies’ or ‘philosophies’ are critically examined. Each ideology contains slippages, and their co-existence can still be regarded as contentious, yet they are repeatedly spoken of in decision-making about maternity care and remain integral to this process.

#### Analytical themes

##### Uncertainty

‘Uncertainty’, along with the following two analytical themes, encompasses several different experiences that are related to mothers’ concerns about the unknown and the course of pregnancy, childbirth and maternity care, all of which shape women’s decision-making and the options they ultimately pursue. Uncertainty about the unknowns of pregnancy and childbirth is most prominent among first-time mothers, who do not have previous experience to inform their choices and may approach care with a level of wariness:

“You don’t know how painful it is going to be, it’s the fear of the unexpected.”[[46] p3].

“The birthing center, I knew it existed, but I didn’t really know. But for a first baby I felt safer at the hospital, because they have all the technology, and if there are problems … I am already at the right place. So, the fear of the unknown, I’d rather be in a place where I know that everything is readily available.”[[45] p163].

Anxiety about having a straightforward pregnancy and birth, fear of pain, and lack of experiential knowledge contribute to women opting for what is viewed to be the safest pathway of care, using what is perceivably less risky to guide their decisions. Uncertainty, which encompasses risk, safety and fear, was frequently cited as an influence on decision-making, regardless of parity. Fear, such as fear of pain, can be a powerful motivator for choosing a specific place of birth:

“Well, of course I am gonna go in and have an epidural, because that’s what the hospital is there for. And of course I am going to take whatever is – I don’t want to handle pain, I’m scared of pain, so I’m definitely going to do it the easy way.”[[50] p644].

Fear of pain is not the only anxiety that mothers experience; some women expressed uncertainty about the safety of the hospital environment, which directed their pregnancy and birth care decisions. Safety and risk are constructed in several different ways in these cases. For some, medical technology, interventions and environments connote safety, security and quality care, while for other, these are view as inherently risky, down to the microscopic level of “germs”:

“You come out of the hospital and you feel like you are covered in germs and you just wanna have a shower and change your clothes so why would I wanna take a newborn who doesn’t have any kind of antibodies in their system yet into a hospital? It didn’t make logical sense to me.”[[53] p579].

“That CTG or that doptone is also based on fear. Yes, then you trust the machine more than what I tell you about how it’s going, or your own intuition. And I understand that you think, as a midwife, you don’t want to be sued, and you don’t want a dead child, and you feel responsible. I understand all that. But it takes away my control over my delivery and my body and what I want.”[[50] p7].

“So, I went on a hospital tour and I said to myself ‘I don’t feel safe here,’ anyone can come in and out and I’m not in control.”[[49] p270].

Fear, risk and safety appear to be part and parcel of uncertainty. Fear about childbirth is strongly connected to the unknown, whether it concerns pain levels, interventions or entire approaches to maternity care, as opposed to concrete calculations of risk, displaying how uncertainty is remains enmeshed with ‘being in control’.

##### Bodily autonomy and integrity

Control, and how it is linked to choice, is a topic widely covered in reproductive and maternal health research. Women frequently discuss their maternity experiences in terms of control, whether it be losing it, maintaining it or reclaiming it. We chose to shift the perspective to bodily autonomy and integrity because ‘control’ is not always explicitly stated by participants, and experiences often refer back to the body. This theme, as with the others, is multifaceted, encompassing a range of experiences. First. the denial of bodily autonomy is commonly coded as “loss of control” by researchers:

“[Y] ou can be in whatever position you want, you can have whatever doctor you want, but then the nurses come in and they tell you to do this, so you do it” (Shirley). Andrea also noted, ‘During prenatal classes, they had shown us some labor positions to try and stuff, but [in hospital] they were like, ‘Oh, just try and rest. Lie down.’“[[50] p645].

“I felt violated and humiliated. It ended up with the doctor telling me my baby was stuck and she would try to pull my baby out, in theatre, with an epidural, surrounded by strangers, in case it didn’t work in which case they would perform an emergency c-section. It was the most awful experience of my life.”[[41] p5].

Experiences in this category may be quite subtle, such as threats of induction or discussions that aim to limit women’s decisions about their bodies and care, to more transparent, like being forced to lie down, too traumatic, in which women are left feeling “violated”. During these experiences, women’s control over their care is restricted or removed completely, and this is embodied through the type of care received and the way it is received. Some women seek to protect this autonomy and integrity through their care decisions:

“… you’ve got control over your environment, you can decide what position you’re in, whether you need something to eat or a bath or a scented candle or, you know, you might want none of those things, you might have time for none of those things … And being somewhere that is familiar and safe and happy and that is not intruded on by other people and their various dramas, positive or negative. And where you can control the cleanliness and the food and anything else, and you can go to your own bed afterwards and … yes. It just feels to me some … more comfortable.”[[37] p62].

“When I contacted the midwife, I had not decided for a home birth yet, I contacted her because I wanted to have a respectful birth, without epidural analgesia, and I wanted a person who respected that. I knew that I was not going to find a healthcare professional who respected me in this way [at the hospital] …” [[61] p19].

The review outcomes, which included 10 studies of home births and one of free birth, suggest that birthing outside of the hospital is a common route women take if they are concerned with control over their bodies and agency in their care. Pursuing home birth, in which limited medicalisation is ideal, can be viewed as a choice that is informed by and reflects women’s desires for autonomy and integrity, and those who choose this type of care often centre their motivations around their bodies.

##### Performing good motherhood

The final analytical theme is ‘performing good motherhood’ and is related to responsibility and risk, which are often ascribed to mothers to manage. [37] This theme revealed a pervasive, gendered cultural norm that ‘good’ motherhood and birth takes place in a hospital with a team of medical professionals. It may be one of the reasons why women planning home births often describe thorough information gathering and risk assessments, and speak about negotiating their care within the purview of biomedicine:

She [participant] used biomedical knowledge to support her view that birth can safely be conducted at home when risks have been excluded by prenatal care. For this purpose she and her husband had searched medical databases to find research on home births. To secure her own low-risk status she had extra examinations done during her pregnancy. She organised her home birth to represent conditions in the hospital as far as she could.[[57] p1117].

Re-creating the home to mimic a hospital reinforces the authoritative position of the medical system and the norm that maternity optimally takes place within it, while simultaneously demonstrating this woman’s responsibility as a mother, despite her decision to birth outside of this system. However, opting for hospital-based and more medicalised care remains the ultimate act of risk management, and, for women choosing these, risk may be spoken of in terms of danger and death, each seen as possibilities in the spaces outside of it:

“I said, ‘Look, don’t worry, I’m not going anywhere there’s no doctors.’ And he [GP] said, ‘Yes, I’m just saying, you know, because you know the chances are … It’s a 40 minute journey [referring to transfer to OU during labour]. Do you want to risk that?’ No! [Laughs] But yeah, that’s all he really said, but he was right … I’m not risking that, I’m not risking the baby’s life or my life.” [[37] p58].

“Why would I want to put my baby in danger? What if something went wrong?”[[52] p712].

Within biomedicine and wider social discourses, pregnancy is constructed as a time of risk and its reduction [70], and childbirth, subsequently, an event that requires not only hospitalisation but also one that acquires a moral subtext, making it difficult for parents choosing different care to escape blame and stigmatisation [71], and adding a ‘burden’ of choice onto women:

I made an early decision to go to hospital to give birth, even though my midwives here were extremely experienced. And I did talk through that with [my midwife] and say that possibly I might decide not to go to the hospital if everything started very quickly … Yes and I wanted that to happen and for the decision to be taken away from me. I craved a home birth, but ‘my niggles of fear’ wouldn’t let me take the risk. An alarm bell goes off in the back of my head that says but what if something happens? … And my midwife said that if you have it in your head (that you don’t feel safe) then it is going to get in the way of the labour taking place and that you need to be in the place where you are going to birth and then it will just happen.[[63] p52].

Coxon and colleagues [37] argue that discourses of responsibility continue to constrain women’s decisions and the way they discuss birth options outside of the norm, and many of women’s reported decision-making experiences uphold this idea.

#### Temporal dimension of decision-making and informed choice

There are several levels in which temporality underpins decision-making and informed choices about maternity care during pregnancy and for birth. On the most immediate level rests a continuum from ‘early pregnancy’ to ‘late pregnancy’, during which options are considered, information gathered and decisions are made. There is not necessarily a pattern of when decisions about maternity care are made along this continuum, and there may not be specific decisions points along it, but rather decisions can build or shift over time. On the other hand, significant influences, such as attending a friend’s home birth early in pregnancy, can provide a more concrete time stamp for a decision shift along this continuum:

“I had a powerful experience when I was early on [in my pregnancy]. It was home birth of my friend’s...I think for me, deciding to go with home birth had somewhat to do with being there at a home birth...That seems worthwhile: I want to be a part of that. I want that. And so early on, we made that decision.”[[55] p175].

“[F] rom the beginning I knew [hospital birth] was the course of events”[[47] p47].

“Just wanted to leave the window open, because I liked the idea of doing it but I wasn’t completely ready to make that decision, and I think as I got further along in my pregnancy, it was easier for me to make that decision.”[[35] p125].

The next level within this temporal dimension of decision-making is constituted from past personal experiences, which encompass before pregnancy, previous birth(s), significant life events and family experiences of the recent past. Like the themes of maintaining bodily integrity, managing uncertainty, and performing good motherhood, it also has the potential to be a highly emotional layer, where memories of trauma or loss reside and shape how decision-making is approached and which care options women and their partners take seriously:

“I don’t want it to go like it went last time. I had quite a traumatic first birth and I had basically a series of events happened and I felt that I had lost control and I hadn’t been informed properly when I was in labour. So, things kind of went out of control. This time round I’m, I’m planning to be at home because that gives me an element of control that I didn’t feel that I had when I was in hospital.”[[46] p6].

“My brother had … well his wife had a baby at home and the baby died … and I think that affects … that sort of affects the family for a long time, you know, anyone in the family who was involved with that or remembers that, you can’t, [home birth is] just a no-no for us.”[[37] p59].

The deepest layer of this temporal dimension of decision-making is the historic past, which often is embedded within women’s perspectives of birth and their decision-making processes. The ‘past’ or ‘old days’ are often used to justify a decision, particularly when it comes to place of birth. This justification occurs along two lines: either it is connected with traditional birthing practices, inherent biological drives or mammalian physiology to strengthen the choice to home or free birth, or it is connected with historical maternal and infant mortality rates to make a case for hospital birth:

“I accepted that like any other mammal, I can give birth so the implicit trust I have in my biology played a fundamental role in this acceptance of birthing alone.”[[41] p8].

“I just know that bad things can happen, like to the baby’s blood or heartbeat or whatever. Birth is really dangerous and women used to die all the time before hospitals, and I guess they still do in poor countries and all that.”[[52] p712].

In conceptualising temporality, it is easy to present it linearly, reflecting how individuals move forward in time, which is not necessarily the case for a cognitive process like decision-making. Instead, women may shift between or draw from different temporal modes when they working through and making a decision.

## Discussion

Though all of the studies we review concerned decision-making and choice, few focused primarily on the *process* of decision-making, as opposed to the influences on it or placed informed choice as a primary research aim. This could be attributed to the artefact of the research methods used, but this limited focus means that clarifying maternal health decision-making is tenuous at times and tracing the development of informed choice in literature published since 1990 is difficult. In their analyses, many of the review sources failed to surface deeper themes within this vein, leaving questions about what we are missing when we talk about women’s decision-making in relation to birth, for instance how social and institutional power might come to be embodied through decisions about care. Ultimately, we found that decision-making is not only dynamic but also a temporal process, in that it is made within a defined period and invokes the past, whether this is personal, familial, social or historical. However, few of the papers reviewed explicitly explored timing of decisions; instead, time was discussed within reported influences on choice.

Different decision-making strategies intersect over the course of pregnancy, how information is collected and scrutinised and how options are weighed within different boundaries. Feelings of satisfaction, anxiety and safety are used as an inner guide for making a decision, demonstrating the extent to which emotional and bodily well-being are considered when choosing significant care options, such as birth setting. Triangulating information about pregnancy and care is common, given the scope of resources available; however, the act of confirming suggests that relationships of trust can still be difficult to manage with the overwhelming sense of risk attached to maternal health.

Within motherhood, there can be a multitude of experiences, narratives and identities that, like information seeking and relationships with choice, are constantly unfolding and in flux, within a dynamic decision-making field of maternity history, uncertainty, birth ideology and care options. Aligning with a birth philosophy, whether consciously or not, is a crucial component of decision making. Generally, birth philosophy is pushed, by researchers and parents alike, into two fundamental camps, in which pregnancy and birth are treated either as a medical event or a natural event. This dichotomy is consistently generated and reproduced within pregnancy and birth discourse, rhetoric and research, reinforcing a binary tendency in analysis that does not realistically reflect the ways women consider maternity. The ‘natural event’ ideology features predominantly in this review because nearly a third of the included studies focused on home birth or free birth exclusively, and this may not be representative of the wider population; however, focused research into non-hospital birth is unsurprising, as this type of decision-making is more ‘visible’ as a process because it is not normative. The ‘medical event’ ideology remains the authoritative narrative of pregnancy and birth, evidenced by women seeking care outside of the norm still rely on biomedical sanctions to do so, and by the fact that many births in Western countries take place in hospital settings.

This review further highlights the importance of the body in maternal health. Embodiment underpins women’s pregnancies and births [19], reflecting the liminality of these events, even if it is not explicitly discussed. Embodied experiences are central to maternity, evidenced by the importance mothers place on bodily autonomy and integrity, and how indicative emotions are within this. Anthropologists have suggested that emotions affect how the body is experienced, and are connected to images of well or poorly functioning social bodies [72]. Good motherhood continues to be heavily associated with concepts of the appropriate maternal body. Research has previously suggested that a fit, pregnant (or post-pregnancy) body is idealised and celebrated, and thus can be seen as a mark of responsible, good motherhood. We would extend this further to include a good maternal body is one that is *safe and informed* as well as fit. A maternal body out of control, uninformed or medically unregulated induces uncertainty, anxiety, judgement and, to a certain extent, distrust, and this, in turn, shapes decision-making about care. The inter-linking actions highlighted in our review may shift emotional and ideological views of pregnancy and birth, and help individuals to calibrate care options to themselves, their families and their environment. These considerations, strategies and actions are all mediated *through* the maternal body, meaning that they should be considered as *embodied*.

Thus, an informed choice in a maternity care context is one that takes into account all the information gathered and is aligned with a woman’s birth philosophy, the aspects of which have been balanced against those of the other options. However, it is moderated by desires to maintain bodily autonomy and integrity, discourses of uncertainty and motherhood performativity, framed temporally and embedded in emotional and spatial considerations. From the research reviewed, it can be hard to pin down to what extent women regularly make informed choices about their care; however, the instances of it are relatively clear when they are articulated, and this is often by women who choose home or free birth. They appear more likely to consider multiple options, opting for those that are deeply connected to their values and their bodies, and research extensively. This may be indicative of medical systems constructed to support one pathway of care, but there is no reason a woman receiving pregnancy care and giving birth in a more normalised settings should not be facilitated to have similar decision-making experiences. In researching women who choose hospital or more medicalised care, their relationships with choice are key to unpacking whether or not their choices are informed, according to public health policy. Our conceptualisation of informed choice is one of many put forward since it became a buzzword in public health, yet it remains the case that, given the persistent power imbalances in biomedicine, it will continue to be defined by policymakers and HCPs. It may, in fact, be that women “are exposed to frameworks of choice rather than being explicitly able to formulate their own choices”. [[73] p414] If this is the case, then researchers need to interrogate the possibilities for making informed choices in such a framework.

### Limitations and further research

The meta-synthesis procedure provides an excellent guide for making sense of qualitative literature and concepts, but it may require a balancing act of what to include and what to exclude, revealing the inherent subjectivity of qualitative methodologies and the importance of reflexivity and transparency concerning how and why choices were made. Our meta-synthesis reviewed 37 sources, which, by some standards, may be considered too large and, therefore, a limitation. There are no strict recommendations on the size of a review; however, researchers should be aware that the framework may be better suited for smaller reviews, given the analysis and synthesis stages. We did consider curtailing the focus of this review to decision-making and informed choice about place of birth, but, ultimately, we felt that our analysis and discussion would benefit from a wider scope, as the findings would be useful for a broader audience in maternal health research.

In conducting this review, we identified three areas for further research. First, more research is needed on decision-making and informed choice among fathers and non-pregnant partners, as there is currently very little. Existing studies suggests that partners feel disembodied from pregnancy and vulnerable during labour and birth [74, 75], and that they do take active roles in defending women’s choices and preparing for birth [76], showing the need for the development of an evidence base to enhance support and inclusion. Second, future research should prioritise generating findings from a wider European context, which would provide a better picture of women’s experiences in these countries and how they are linked to the health care systems. Much of the research reviewed was based in the UK or USA, and the few studies from Denmark, Finland and Spain provided fresh insights into the national maternity services and decision-making realities within them. Finally, given the limited acknowledgement of the body in the studies reviewed and the dearth of qualitative research on maternal health incorporating theories of embodiment in general [77], future studies on decision-making and informed choice in these contexts should consider this dimension as a key theoretical starting point.

### Implications for policy and practice

Our review demonstrates the need for a reconceptualisation of decision-making and informed choice as it concerns maternity care. Attempting to bridge policy and practice gaps through choice is common in maternity care, but there is often limited reflection on our own assumptions about the fundamental nature of making one, or what constitutes *informed* choice. Historically, women’s choices have been constrained, particularly concerning their livelihoods, and we should be mindful of how gender and hierarchy are iterated by contemporary frameworks of choice, such as through the performance of good motherhood. There is little consideration of the embodied dimensions of pregnancy, birth and decision-making experiences in classical theories of choice, or cyclical notions of temporality in current decision-making models, such as SDM. Reid et al. [17] similarly found the framework for women’s decision-making processes about antenatal screening generated from their meta-synthesis was not fully congruent with existing theoretical frameworks. Our synthesis highlights informed choice as layered within a multidimensional process of decision-making, which may not be straightforwardly achieved through enshrinement in policy alone.

Our findings indicate that health systems should more actively incorporate flexibility and fluidity into practice, which was highlighted by several of the studies reviewed, but this may conflict with rigid structures for care. Maternal health experiences are highly personal, and there is need for care systems that are flexible enough to accommodate them. However, hospital care has been compared to an industrial factory, in which labour care resembles an assembly line [78, 79], and further research suggests that funnelling through specific care is still occurring. [80–82] Inflexible information provision and care pathways may account for why women continue to rely on social and familial networks and different media for support. They provide opportunities to acquire key maternity knowledge but also leave space for personalisation, in that women are free to engage with information when and as they choose. Decisions about care do not always take place at fixed points in time, and this must be considered within any maternity choice framework.

In thinking of solutions, it is more important than ever to embrace emergent models in maternity, like midwifery-led care, continuity of carer or group antenatal care [83], which support more fluid decision-making, break down dichotomies and facilitate the social network effect [84, 85]. However, given the slow development of these models in health systems and comparatively low uptake of ‘alternative’ options among women, even in countries with strong policy and midwifery integration [86], there is still significant work to be done on normalising these pathways. HCPs, given their trusted expertise, hold a unique position, allowing them to spearhead change in maternity care, beginning by shifting narratives of safety and risk. Women are often concerned with uncertainty in pregnancy, labour and birth when making a decision, so effectively and transparently communicating about risk and safety in different care settings will be crucial in promoting and legitimising emergent models. Placing more trust and respect, especially trusting women’s experiences and respecting their bodily autonomy and integrity, in care giving and receiving will also help to make strides in restructuring not only the services, but the way we think about relationships within them.

## Conclusions

This is the first systematic review to take a wider maternity context into consideration in order to elucidate decision-making and informed choice about pregnancy and birth care. Building on the critical reinterpretation of the included studies, we developed a conceptual synthesis, which was consistent with the findings of these but also extended beyond them. Most of the studies reviewed here were of moderate to high quality, and there has been an increasing focus on decision-making and informed choice, particularly in regards to place of birth, in the literature since the 2010s, a trend that is likely to continue, as choice remains prominent in public health policymaking. In light of this review, we urge researchers, practitioners and policymakers to take the embodied experience of pregnancy and birth and dynamic processes of decision-making into account when forming care pathways and tackling informed choice therein.

## Data Availability

The datasets used and/or analysed during the current study are available from the corresponding author on reasonable request.

## Abbreviations

CASP: Critical Appraisal Skills Programme
HCP: Health care professional
NHS: National Health Service
PRISMA: Preferred Reporting Items for Systematic Reviews and Meta-Analyses
PROSPERO: International prospective register of systematic reviews
RCT: Randomised controlled trial
SDM: Shared decision-making
UK: United Kingdom
USA: United States of America
